# Functional analysis of differentially expressed circular RNAs in sheep subcutaneous fat

**DOI:** 10.1186/s12864-023-09401-6

**Published:** 2023-10-05

**Authors:** Tian-yi Liu, Hui Feng, Salsabeel Yousuf, Ling-li Xie, Xiang-yang Miao

**Affiliations:** State Key Laboratory of Animal Biotech Breeding, Institute of Animal Science, Chinese Academy of Agricultural Sciences (CAAS), Beijing, 100193 China

**Keywords:** Sheep, CircRNA, Subcutaneous fat, Fat deposition, Lipid metabolism

## Abstract

**Background:**

Circular RNAs (circRNAs), as important non-coding RNAs (ncRNAs), are involved in many biological activities. However, the exact chemical mechanism behind fat accumulation is unknown. In this paper, we obtained the expression profiles of circRNAs using high-throughput sequencing and investigated their differential expression in subcutaneous fat tissue of Duolang and Small Tail Han sheep.

**Results:**

From the transcriptomic analysis, 141 differentially expressed circRNAs were identified, comprising 61 up-regulated circRNAs and 80 down-regulated circRNAs. These host genes were primarily enriched in the MAPK and AMPK signaling pathways which is closely associated with fat deposition regulation. We identified *circRNA812*, *circRNA91*, and *circRNA388* as vital genes in fat deposition by miRNA-circRNA target gene prediction. The functional annotation results of target genes of key circRNAs showed that the signaling pathways mainly included PI3K-Akt and AMPK. We constructed the competing endogenous RNA (ceRNA) regulatory network to study the role of circRNAs in sheep lipid deposition, and *circRNA812*, *circRNA91*, and *circRNA388* can adsorb more miRNAs. *NC_040253.1_5757*, as the source of miRNA response element (MRE) among the three, may play an important role during the process of sheep fat deposition.

**Conclusions:**

Our study gives a systematic examination of the circRNA profiles expressed in sheep subcutaneous fat. These results from this study provide some new basis for understanding circRNA function and sheep fat metabolism.

**Supplementary Information:**

The online version contains supplementary material available at 10.1186/s12864-023-09401-6.

## Introduction

Meat is an important part of human diet. With the improvement of people's living standards, the demand for the taste and nutritional value of meat is increasing. Cultivating high-quality meat has been the goal of livestock industry. Sheep are the main livestock resources of meat, milk, and fur in the world. Compared with other livestock and poultry, mutton is fresh and tender, with unique flavor and high nutritional value [1]. In animal production, the deposition of fat in different parts affects the meat production performance, muscle quality and nutritional value. There are certain differences in the metabolism of fat and the molecular regulation mechanism in different parts. Fat can be divided into subcutaneous fat, intramuscular fat and visceral fat according to different anatomical sites [2]. Subcutaneous fat tissue accounts for the highest proportion of total fat content in animals [3], and has the function of protecting animals and storing energy. In addition, subcutaneous fat tissue is better at absorbing circulating free fatty acids and triglycerides [4]. The subcutaneous fat content had a moderate correlation with the intramuscular fat content. This provides a basis for exploring the regulatory mechanism of fat content in different parts of the body, which can be independent of each other [5]. The intramuscular fat content can easily affect the edible and nutritional value of mutton [6]. To meet the market demand, the breeding industry paid more attention to the growth rate of sheep, which led to poor meat quality. In fact, problems of meat quality related to fat and human health related to lipid metabolism have attracted extensive attention. Studies have found that ncRNAs are involved in the regulation of fat development.

MiRNAs have been confirmed to regulate the expression of genes related to adipogenic differentiation and lipid metabolism. For example, *miR-143*, a regulatory role in the adipogenic differentiation, was first found to promote adipogenic differentiation by inhibiting the expression of *MAP2K5* [7]. *MiR-244* regulates the adipogenic differentiation of bovine precursor adipocytes by targeting *LPL*. When *miR-224* was overexpressed, the mRNA expression levels of the markers related to lipogenesis, *PPARγ*, *FASN*, *C/EBPα*, *C/EBPβ*, and *PLIN1* were decreased. In addition, when *miR-224* was inhibited, the opposite effect occurred [8]. CircRNAs can also act as molecular sponges for miRNAs, competitively binding miRNAs and inhibiting the impact of miRNAs on mRNAs translation function. Therefore, circRNAs can indirectly regulate the expression of downstream target genes [9, 10].

The circRNA was the first type of closed-loop RNA molecule found in virus-like samples [11, 12]. However, it did not attract much attention. Until 1979, Hus et al. [13] found that circRNA was the product of endogenous RNA generated by special splicing. With the continuous development of sequencing technology, tens of thousand circRNAs have been discovered. Moreover, circRNA was more stable than linear RNA because it had no 5' cap and 3' poly (A) tail structure and cannot be degraded by ribonucleases [14]. CircRNAs have various functions and have become markers of disease [15, 16]. Sang et al. [17] found that *hsa_circ_0025202* can act as miRNA sponge of *miR-182-5p* to further regulate the expression and activity of *FOXO3a*, thereby playing an anti-cancer role in HR-positive breast cancer. In general, circRNAs were expressed at low levels [18]. Existing studies have shown that circRNAs can participate in fat deposition and lipid metabolism through miRNAs.

*CircSAMD4A* was a gene with significant difference in clinical samples of fat between thin and obese individuals. Studies in mice found that *circSAMD4A* regulated preadipocyte differentiation by acting as *miR-138-5p* sponge, thereby increasing *EZH2* expression. This indicates that *circSAMD4A* can be a potential target in the treatment of obesity-related metabolic diseases [19]. Li et al. [20] analyzed circRNA in subcutaneous fat tissues of Large White and Laiwu pigs with differences in genetic background. They found that *circRNA_26852* and *circRNA_11897* were involved in lipid metabolism as miRNA molecular sponges. Shen et al. [21] found that *circINSR* overexpression significantly promoted myoblast and preadipocyte proliferation and inhibited apoptosis. In addition, *circINSR* inhibited preadipogenesis by reducing *mir-15/16* inhibition of target genes *FOXO1* and *EPT1*. Similar results were found for donkeys [22] and chickens [23]. However, the molecular mechanisms of circRNAs regulation sheep fat deposition remain largely unknown.

This study focuses on the role of the circRNA in fat deposition, so the greater difference in genetic background, the more differentially expressed genes are obtained from the screen. Inferring gene function based on functional enrichment results. Duolang sheep and Small Tail Han sheep are high-quality sheep breeds. Among them the Duolang sheep has a high capacity for fat deposition and is an excellent dual-purpose sheep breed for meat and fat, representing an excellent sheep breed in Xinjiang [24]. Duolang sheep can deposit a large amount of fat in the body to meet nutritional demands during the winter and spring [25]. The Small Tail Han sheep is a representative of the Mongolian sheep, a short and thin-tailed sheep with strong reproductive ability. The fat is mainly distributed around the kidney to adapt to the harsh climatic conditions [26]. Since there are differences in fat deposition between Duolang sheep and Small Tail Han sheep, RNA-Seq was used to identified the expression patterns and potential functions of circRNAs in sheep subcutaneous fat tissues. This provides a theoretical basis for further research on the regulatory role of circRNAs in lipid metabolism.

## Materials and methods

### Experimental animals and sample preparation

Experimental animals included Duolang sheep and Small Tail Han sheep with the difference in fat deposition. Three female sheep of each breed were used. The feeding, housing, and environmental conditions are control variables in the experiment. All sheep were fed a diet that met their current nutritional requirements. They can freely drink and eat under natural light. At slaughter age (2 years old), the weights of the species were similar (50 ± 3 kg), and all were healthy and in good physical condition. Animals were slaughtered in accordance with Agricultural Industry Standard of the People's Republic of China (NY/T 3469–2019). Animals were stunned with electricity and slaughtered by hanging. The fur was peeled off, and the back fat was used as the material. We collected the fourth rib from the bottom of the sheep. The subcutaneous fat tissue was sampled into a 5 mL tube within 30 min after slaughter and immediately frozen in liquid nitrogen. Then, the sample was transferred to freezer at − 80 ℃ for the long-term preservation and further total RNA extraction.

### Total RNA isolation and quality control

Put approximately 100 mg of sample into a mortar and grinded. The total RNA was extracted from the fat using TRIzol (Invitrogen Life Technologies, Carlsbad, USA). The genomic DNA was removed using rDNase I RNase-free (TaKara, Japan)). RNA quality was verified using a 2100 Bioanalyzer (Agilent Technologies, Santa Clara, CA, USA) and the NanoDrop 2000 (Thermo Scientific, USA). Only high-quality RNA samples (OD260/280 = 1.8 ~ 2.2, OD260/230 ≥ 2.0, RIN ≥ 8, 28S: 18S ≥ 1.0, > 10 μg) were used to construct sequencing libraries.

### Library preparation and RNA sequencing

The circRNA and mRNA library were constructed using the TruSeqTM Stranded Total RNA Kit (Illumina, San Diego, CA). And the ribosomal RNA (rRNA) was removed from 2 μg total RNA by the Ribo-Zero Magnetic kit (EpiCentre Biotechnologies, Madison, WI, USA). The RNA was fragmented and reverse-transformed to synthesize cDNA, then the adaptor was ligated. The cDNA second strand was digested with UNG enzyme, amplified by polymerase chain reaction (PCR), and purified to obtain the final library. The miRNA library was constructed using the TruSeqTM Small RNA sample prep Kit (Invitrogen) according to the instructions. After removing the rRNA, the 3' and 5' end adapters were connected to the kit separately, and then the primers were reversed and PCR cycles were performed. Next, the library was enriched, purified and quantified [27]. At last, high-throughput sequencing was conducted using the Illumina NovaSeq 6000 ( Supplementary Fig. 1).

### CircRNA and miRNA sequencing analysis

After the sequencing data were obtained, we performed quality control using Fastp [28] to eliminate sequences, including sequencing junctions, low-quality reads, high N rate and excessively short length. Thus, the high-quality clean data was obtained to ensure the accuracy of subsequent analysis results. The reference species was sheep (Ovis_aries), and the reference genome (GCF_002742125.1, https://www.ncbi.nlm.nih.gov/genome/83?genome_assembly_id=351950) was from the NCBI database [29]. We compared the results after quality control with the reference genome to obtain mapped reads. In addition, the quality of the comparison results of transcriptome sequencing was evaluated. We compared the clean reads of longRNA-seq with the reference genome using the comparison tool Hisat [30], and performed mapping statistics. Since the reads at the trans-shear position of the circRNA cannot be directly compared with the genome, based on Back splice junction (BSJ) reads, we compared the clean data with the CIRI2 [31] and FindCirc [32]. The unions of the two were used for circRNA prediction [32] and compared with the database circBase (animals). Using RPM as the quantitative indicator. After the expressions were obtained, the differential expressions of circRNAs were analyzed the DESeq2 statistical analysis. Screening was performed using |Fold Change|≥ 2 and *P-*value < 0.05 as the thresholds. Then, the numbers of circRNAs up/down were obtained. Based on our previous results on miRNAs, the screening conditions for differentially expressed miRNAs were also |Fold Change|≥ 2 and *P-*value < 0.05 [27].

### CircRNA-miRNA-mRNA regulatory network

DESeq2 was used for differential expression analyses of circRNAs and miRNAs. The |Fold Change|≥ 2 and *P-*value < 0.05 was used to screen the differentially expressed circRNAs and miRNAs from Duolang and Small Tail Han sheep. CircRNAs can act as sponge absorbers of miRNAs to regulate gene expression [33]. The circRNAs in each tissue list were named by assigning consecutive unique numerical identifiers. Differentially expressed circRNAs were screened for target relationship between miRNAs and circRNAs using miRanda. The predicted relationship with the Score was greater than 160 and Energy was less than -20. The interaction network between circRNAs and miRNAs was constructed using Cytoscape V.3.9.1 [34]. In general, the nodes with many edges are key genes. Many nodes are important. To further study the functions and interactions among key circRNAs and miRNAs, the miRanda software (http://www.miranda.org/) [35] was used to predict the target genes of differentially expressed miRNAs (Score ≥ 160 and Energy ≤ -20). After predicting the target genes of miRNAs, we mapped the targeting relationship between circRNA-miRNA-mRNA using Cytoscape software.

### Sequence analysis of key circRNA

After screening out the key circRNAs, we analyzed the sequence of the key circRNAs through the online tool ORF Finder [36] (http://www.bioinformatics.org/sms2/orf_find.html), and obtained the prediction results of open reading frames (ORF). Then, we inputted the prediction results into the Conserved Domains database (https://www.ncbi.nlm.nih.gov/Structure/cdd/wrpsb.cgi) to predict the coding protein ability of the ORFs of circRNAs.

### GO and KEGG enrichment analysis

We conducted Gene Ontology (GO) enrichment analysis on source genes of differentially expressed circRNAs through Goatools [37]. The obtained functional annotations of genes included three parts: biological process, molecular function and cellular component [38]. Similarly, the host genes were enriched by Kyoto Encyclopedia of Genes and Genomes (KEGG) pathway [39], and the genes were enriched by metabolic and information processing pathways by R. V.4.2.1 [40]. It was statistically significant when the *P-*value was small. The corrected *P-*value was obtained by BH (Benjamini and Hochberg). It was significant enrichment at corrected *P-*value < 0.05. After obtaining the functional enrichment of the source genes, we conducted the functional enrichment analysis on the target genes of the candidate genes.

### Analysis of mRNA clustering, structure and subcellular localization

The expression patterns of the circRNA target genes were clustered and analyzed after the screening. We calculated the distances between the genes and the samples based on the information about the expression of genes in different samples. Then, the genes and the samples were classified through the iterative method, respectively. The algorithms of genes were hierarchical clustering, the clustering method was the complete linkage [41]. This can visualize the gene expression in each sample. Genes with similar expression patterns usually have functional correlation, and the function of unknown genes can be inferred [42]. The protein motif was predicted through online tool MEME [43] (https://meme-suite.org/meme/doc/meme.html), and the motif information was obtained by inputting the CDS region of mRNA. Through online software of EUK-MPCOC [44] (http://www.csbio.sjtu.edu.cn/bioinf/euk-multi-2/), we predicted the subcellular localization of eukaryotic proteins, and studied the cellular localization information of protein to predict its biological functions.

### Quantitative real-time polymerase chain reaction

Quantitative real-time polymerase chain reaction (qRT-PCR) was used to verify the reliability of the sequencing results. We mixed three biological duplicate samples, and three biological replicates were employed for each gene. Total RNAs were reverse transcribed to synthesize cDNAs using GeneAmp® PCR System 9700 (APPlied Biosystems, USA). QRT-PCR was carried out using the BIOER LineGene 9600Plus fluorescent quantitative PCR assay, and the PerfectStartTM Green qPCR SuperMix kit on a LightCycler® 480 II fluorescent quantitative PCR assay (Roche, Swiss). With *GAPDH*, *ACTB*, and *U6* as internal references of circRNA, mRNA, miRNA, respectively. The expression levels were calculated by 2^−∆∆Ct^ method [45]. The relative expression levels were analyzed using t-test statistics. The corresponding primer sequences are shown in Supplementary Table 1.

#### Statistical Analysis

All data were presented as means ± SDs. The Student’s t-test was performed for comparison by Excel (Microsoft 2016), and *P-*value < 0.05 was statistically significant.

## Results

### Summary of circRNA sequencing results

To study the difference in the subcutaneous deposition of fat tissue between Duolang sheep and Small Tail Han sheep, we performed high-throughput sequencing on the fat of the two breeds of sheep. In this study, Duolang sheep and Small Tail Han sheep were divided into group D and group X. In this experiment, a total of 113.29 Gb clean data was obtained from six samples. The clean data in each sample reached more than 17.84 Gb, the percentage of Q20 bases was above 98.30%, and the percentage of Q30 bases was above 95.01% (Table 1). After quality control and comparison with the reference genome, the mapping rate of each sample was above 94.69%. This indicates that the reference genome was fully annotated, and there was no pollution in the experiment. This provides a good foundation for the subsequent data analysis (Table 2).

**Table 1:** Quality control statistics of sequencing data for each sample

Sample	Raw Reads	Raw Bases	Clean Reads	Clean Bases	Error rate(%)	Q20(%)	Q30(%)	GC content(%)
D-PF-1	136,631,686	20,631,384,586	135,393,598	18,909,175,613	0.02	98.44	95.22	52.05
D-PF-2	141,871,442	21,422,587,742	140,587,412	19,452,612,042	0.02	98.30	95.01	52.78
D-PF-3	144,353,224	21,797,336,824	142,895,188	19,940,935,825	0.02	98.44	95.23	52.09
X-PF-1	137,953,526	20,830,982,426	136,746,686	19,091,441,640	0.02	98.50	95.38	52.25
X-PF-2	129,535,766	19,559,900,666	128,433,640	18,053,934,666	0.02	98.40	95.20	52.45
X-PF-3	132,333,836	19,982,409,236	130,999,204	17,840,367,245	0.02	98.41	95.17	52.23

**Table 2:** Reference genome comparison results for each sample

Sample	Total reads	Total mapped	Multiple mapped	Unique mapped
D-PF-1	135,393,598	130,223,053(96.18%)	31,002,401(22.90%)	99,220,652(73.28%)
D-PF-2	140,587,412	133,116,845(94.69%)	26,205,549(18.64%)	106,911,296(76.05%)
D-PF-3	142,895,188	137,830,324(96.46%)	34,303,949(24.01%)	103,526,375(72.45%)
X-PF-1	136,746,686	131,380,772(96.08%)	29,846,253(21.83%)	101,534,519(74.25%)
X-PF-2	128,433,640	123,830,160(96.42%)	28,079,034(21.86%)	95,751,126(74.55%)
X-PF-3	130,999,204	125,839,893(96.06%)	28,446,958(21.72%)	97,392,935(74.35%)

### CircRNA characterization

**Fig. 1:**
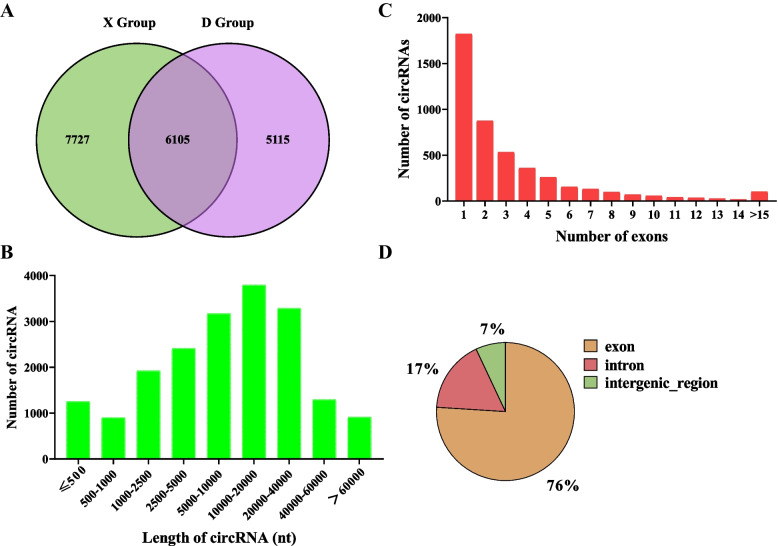
General Characteristics of circRNAs in the Sheep **A** Number of circRNAs identified in Duolang sheep (D Group) and Small Tail Han sheep (X Group). **B** Distribution of circRNAs in fat of sheep. The X axis represents the length distribution of circRNAs, and the Y axis represents the number of circRNAs. **C** Distribution of the number of circRNA exons. The X axis represents the number of exons, and the Y axis represents the number of circRNAs. **D** Classification of circRNA in subcutaneous fat tissue of sheep

In this experiment, 11,220 and 13,832 circRNAs were obtained from the subcutaneous fat tissues of Duolang and Small Tail Han sheep, respectively. We identified 18,947 circRNAs in total from 6 subcutaneous fat (Supplementary Table 2). 6,105 circRNAs were co-existed in two breeds of sheep (Fig. 1A). The statistical results of circRNA lengths are shown in Fig. 1B. The statistics of circRNA exons showed that most circRNAs consisted of 1 to 3 exons (Fig. 1C). Similarly, the majority of circRNA types were exon (75.69%). This was consistent with the study of others [46], followed by intronic (17.48%), and some types were intergenic_region (6.83%). One end of the starting point or termination point was located in the intergenic region, or both the starting point and termination point were located in the exonic region of a gene, but both exons were not in the same gene (Fig. 1D). This indicates that the circRNA we obtained has no impurities.

### Differential expression analysis of circRNA

**Fig. 2:**
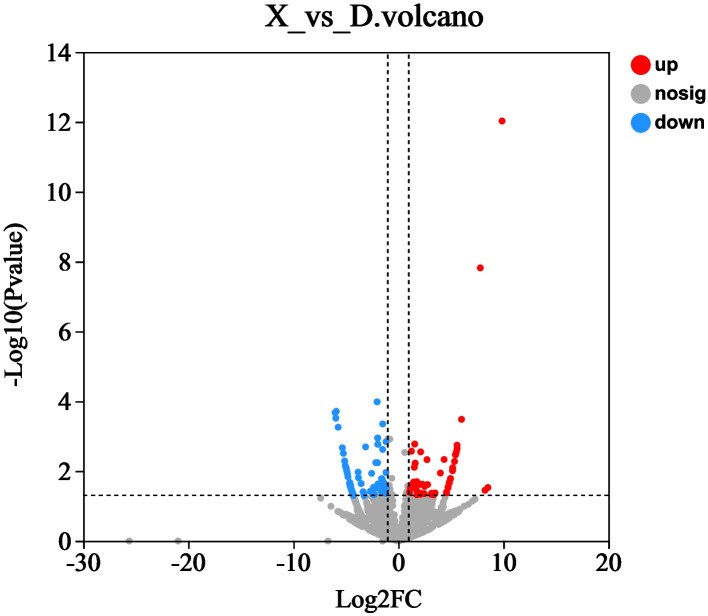
The volcano. The X-axis represents Log2(Fold Change); The Y-axis represents -log10 (*P-*value); Red dots indicate significant up-regulation, blue dots indicate significant down-regulation, and gray indicates no significant difference

We performed the differential analysis of circRNAs to examine the expression patterns of circRNAs in the fat deposits of two breeds of sheep.A total of 18,947 circRNAs were identified, and their RPM values were calculated. The criteria for screening differentially expressed genes were |Fold Change|≥ 2 and *P-*value < 0.05. The results showed that 141 differentially expressed circRNAs were identified, including 61 up-regulated circRNAs in Duolang sheep and 80 down-regulated circRNAs (Fig. 2 and Supplementary Table 3). Among the differentially expressed circRNAs, 29 were only expressed in the Duolang sheep, and 45 were only expressed in Small Tail Han sheep. The results showed that circRNAs were different in the lipid deposition of the two sheep.

### Relationship between circRNA and miRNA

**Fig. 3:**
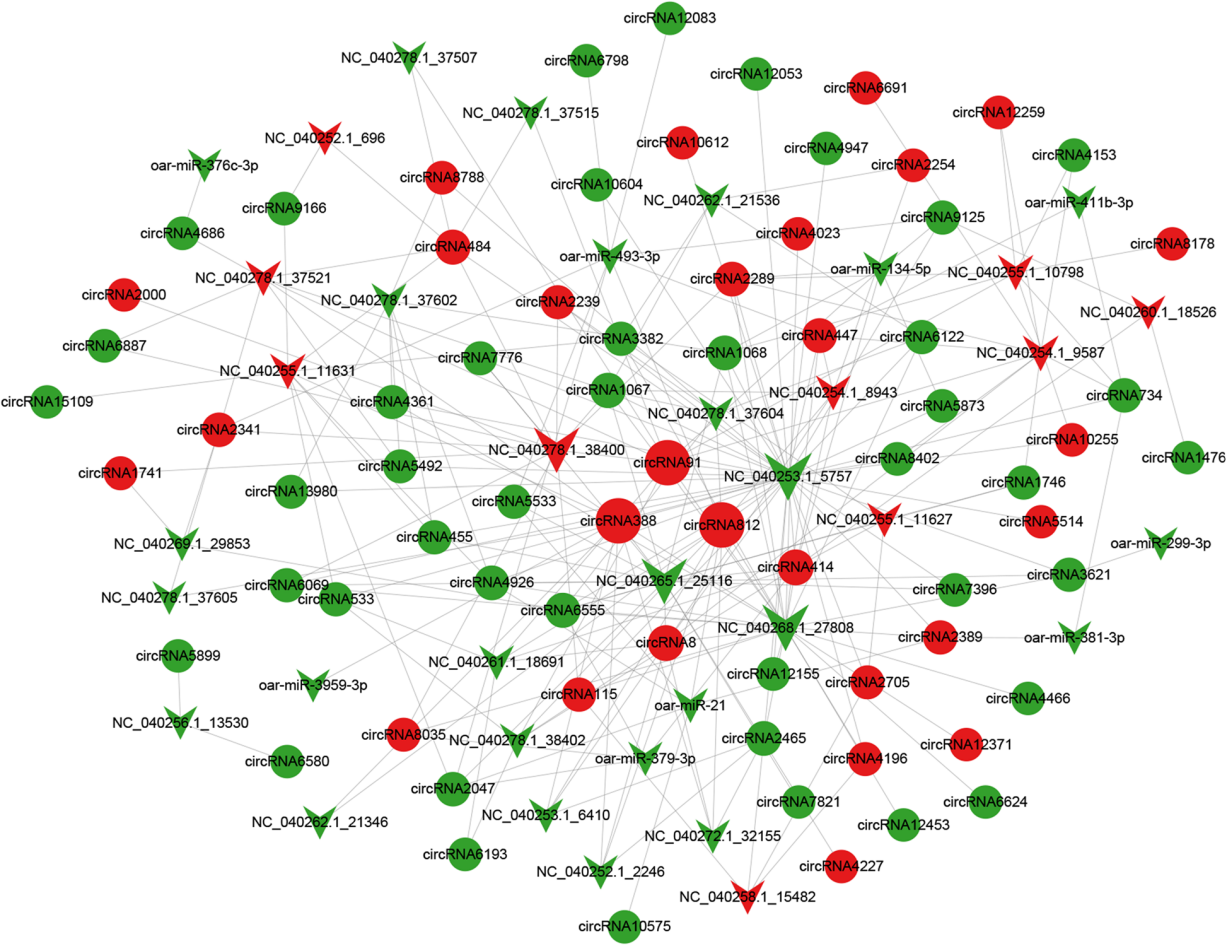
Regulatory networks of genes. CircRNA-miRNA regulatory network in sheep subcutaneous fat tissue. The circle represents circRNA, and the arrow represents miRNA, The red represents up-regulated expression in Duolang sheep, and the green represents down-regulated expression. Node size indicates degree

To further study the differential mechanism of fat deposition caused by circRNAs, we predicted the targeting relationship between circRNAs and miRNAs. According to the prediction of miRanda, there were 236 targeting relationships of miRNA-circRNA, including 36 miRNAs and 71 circRNAs (Fig. 3). By mapping the targeting relationship between circRNA and miRNA into a network diagram, it can be more intuitively discovered that the degree of *circRNA812*, *circRNA91* and *circRNA388* rank the top three. These can be used as molecular sponges for more miRNAs, and all of them were up-regulated in the subcutaneous fat tissue of Duolang sheep. In the ceRNA network, there was an up-down-up mode. A total of 17 miRNAs were predicted to be down-expressed. *NC_040253.1_5757* was the highest degree among the miRNAs, with a targeting relationship with three circRNAs, and were down-regulated in Duolang sheep. Therefore, *circRNA812*, *circRNA91* and *circRNA388* may be the key genes responsible for the difference in fat deposition.

### Sequence analysis of key circRNA

ORF is a sequence that may encode protein in the genome of an individual organism, located between the start codon and the stop codon. Part of circRNAs can encode proteins. Therefore, this paper predicted the sequences of *circRNA812*, *circRNA91* and *circRNA388*. *CircRNA812* was located on chromosome NC_040253.1 of sheep. The chain was positive, belonging to exon, and the shear position was GT-AG. The ORF prediction results show that *circRNA812* contained a total of 221 ORFs without conservative domains. The *circRNA91* was located in the chromosome NC_040271.1 of sheep. The chain was positive, belonged to intergenic_region, and the shear position was GT-AG. The ORF prediction results show that *circRNA91* contained 345 ORFs. The Conserved Domains potential coding ability of these ORFs was predicted, as shown in Table 3. The *circRNA388* was located on chromosome NC_040259.1 of sheep. The chain was positive, belonging to exon, and the shear position was GT-AG. The ORF prediction results show that the *circRNA388* contained 232 ORFs. The prediction results of coding capability were shown in Table 4. *circRNA812*, *circRNA91* and *circRNA388* had potential coding ability.

**Table 3:** CircRNA91 encoding capability prediction information

Query	Hit type	PSSM-ID	From	To	E-Value	Bitscore	Accession	Short name
Q#169	superfamily	413,402	26	88	4.38E-12	61.81	cl02614	SPRY superfamily
Q#383	superfamily	413,489	10	71	1.31E-08	51.52	cl02808	RT_like superfamily
Q#681	specific	409,495	15	106	9.99E-60	178.19	cd07698	IgC1_MHC_I_alpha3

**Table 4:** CircRNA388 encoding capability prediction information

Query	Hit type	PSSM-ID	From	To	E-Value	Bitscore	Accession	Short name
Q#233	superfamily	416,404	14	59	1.04E-15	67.27	cl12015	Adenylation_DNA_ligase_like superfamily
Q#275	superfamily	416,404	10	33	2.28E-06	41.07	cl12015	Adenylation_DNA_ligase_like superfamily
Q#349	superfamily	413,489	18	50	2.52E-08	47.67	cl02808	RT_like superfamily
Q#355	superfamily	400,347	45	139	0.0030888	37.30	cl06820	Toxin_trans superfamily

### GO and KEGG enrichment analyses

**Fig. 4:**
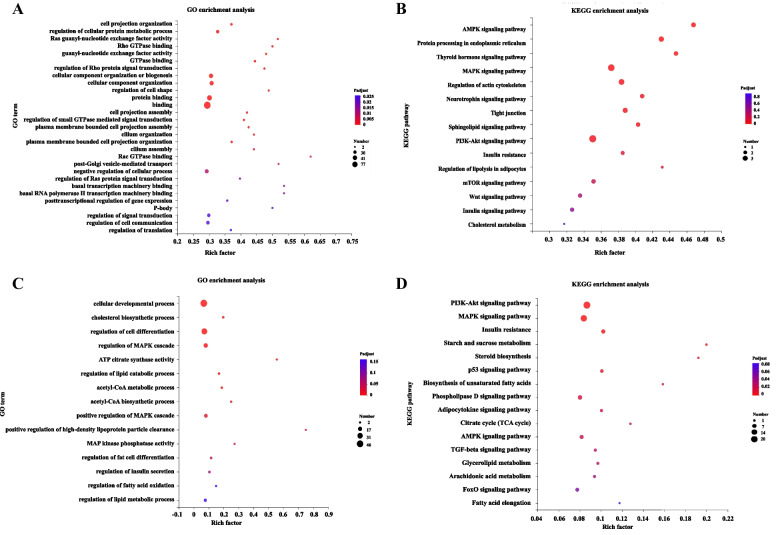
**A** GO enrichment analysis and **B** KEGG pathway analysis of circRNA source genes. **C** enrichment analysis and **D** KEGG pathway analysis of the target genes of circRNAs

Fat deposition is the result of a combination of adipocyte differentiation, proliferation, lipid metabolismand and lipid transport, and is closely related to genetic background, developmental stage and nutritional level. To explore the regulatory mechanism of differentially expressed circRNAs, we performed GO and KEGG enrichment analysis on host genes and target genes of differentially expressed circRNAs in the two groups. First, by analyzing the host genes of circRNAs, we found that the differentially expressed circRNA host genes mainly enriched in the processes of cell projection organization and protein binding (Fig. 4A). However, in KEGG pathway, the host genes are significantly enriched in AMPK signaling pathway, MAPK signaling pathway, and sphingolipid signaling pathway (Fig. 4B). We hypothesized that these circRNAs may play an important regulatory role during the process of fat deposition.miRanda was used to predict target genes for 17 miRNAs, and the results showed that there were 472 target genes were obtained (Supplementary Table 4). The target genes of circRNAs were analyzed for GO and KEGG pathway enrichment. And the target genes were significantly enriched in the GO term, such as cholesterol biosynthetic process, regulation of MAPK cascade, and regulation of fat cell differentiation (Fig. 4C). KEGG was significantly enriched in PI3K-Akt signaling pathway, MAPK signaling pathway, AMPK signaling pathway and TGF-beta signaling pathway (Fig. 4D). Therefore, circRNAs can regulate fat through the above pathways.

### mRNA clustering, structure and subcellular localization

**Fig. 5:**
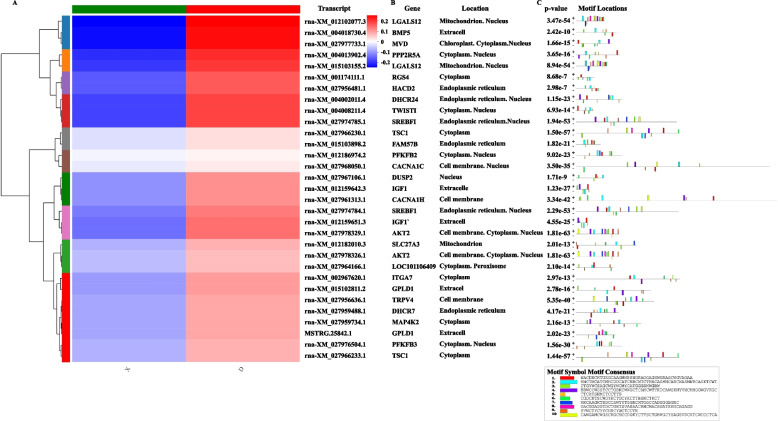
Target mRNAs of subcutaneous fat tissue associated with circRNAs. **A** Each column represents a group, and each row represents a transcription. The red represents a higher expression level of the transcription in the group, and blue represents a lower expression level. The specific expression size trend is shown in the upper left color bar. On the left is the transcription cluster tree. The closer the branches are to each other, the transcript name is in the middle, and the corresponding gene name is on the right side. **B** Subcellular localization of all target genes. **C** Prediction of protein motif localization

miRanda software was used to perform miRNAs target prediction for circRNAs. In combination with the results on miRNAs and mRNAs in subcutaneous adipose tissues of Duolang and Small Tail Han sheep. According to the results of functional enrichment of mRNA, we screened out the parts related to fat deposition as the final target genes. To further understand the structure and function of the target mRNAs, we performed clustering analysis (Fig. 5A) and comparative analysis of subcellular localization (Fig. 5B) on target mRNAs related to fat deposition. In addition, we performed protein motif prediction (Fig. 5C) on CDS regions of these genes. The results show that the branches of gene *LGALS12*, *BMP5*, *MVD*, *PPP2R5A*, *LGALS12*, *RGS4*, *HACD2*, *DHCR24*, *TWIST1* and *SREBF1* were close. It was proved that their expression levels were close, and they are all highly expressed in Duolang sheep. These genes played an important regulatory role in the deposition of fat tissues in two breeds.

### CircRNA-miRNA-mRNA network

**Fig. 6:**
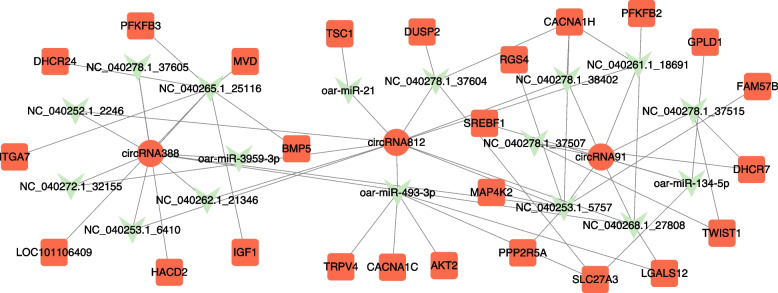
circRNA-miRNA-mRNA interaction networks. Red circles represent up-regulated circRNAs in Duolang sheep subcutaneous fat tissue. Green arrows represent down-regulated miRNA in Duolang sheep subcutaneous fat tissue. Red squares represent up-regulated mRNA in Duolang sheep subcutaneous fat tissue

Competing endogenous RNA (ceRNA) regulates mRNA at the post-transcriptional level by binding to a shared miRNA binding site [47]. When miRNAs are competitively bound by circRNAs or lncRNAs, the levels of mRNAs transcription regulated by the miRNAs are altered and align with the expression trend of circRNAs or lncRNAs. There was an up-down-up regulation pattern between circRNA-miRNA-mRNA. To further study the regulatory mechanism of circRNAs, we constructed a circRNA-miRNA-mRNA network of target genes that were enriched in items related to fat deposition (Fig. 6). We constructed ceRNA networks of *circRNA812*, *circRNA91* and *circRNA388*. They served as molecular sponges for *NC_040253.1_5757*. Functional enrichment analysis showed that *PPP2R5A* was an important gene in the PI3K-Akt signaling pathway and AMPK signaling pathway. Therefore, *PPP2R5A* is closely related to the fat deposition process. *circRNA812*, *circRNA91* and *circRNA388* are important regulatory genes of fat deposition in sheep.

### qRT-PCR

**Fig. 7:**
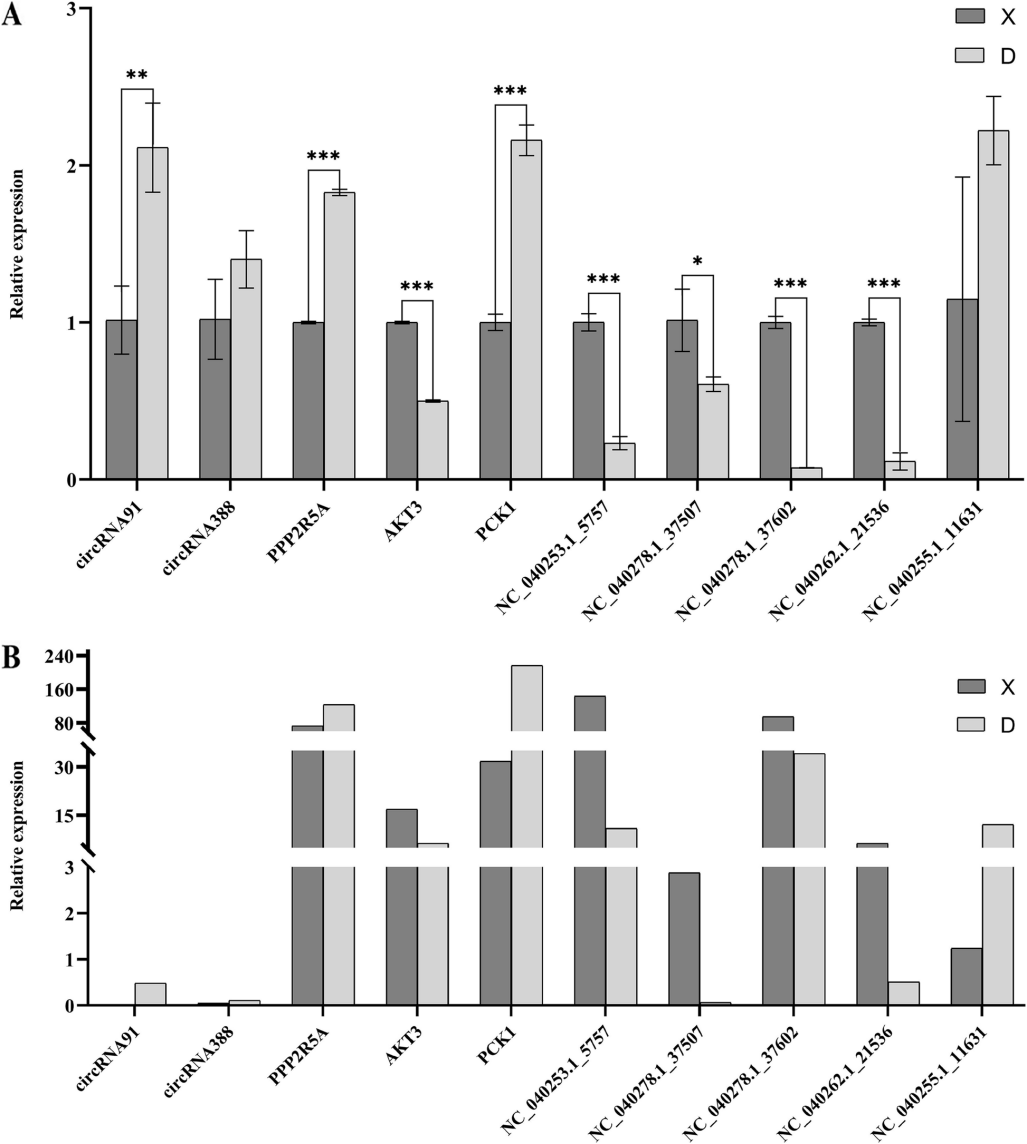
Validation of RNA-Sequencing (RNA-Seq) data using qRT-PCR. **A** qRT-PCR, **B** RNA-seq.The qRT-PCR validation of differentially expressed circRNAs, miRNAs and mRNAs. The abscissa is differentially expressed RNAs, the ordinate is relative expression. The dark is group D, light is group X. Note: *: *P* < 0.05; **: *P* < 0.01; ***: *P* < 0.001

To verify the accuracy of the RNA-Seq data, we examined the relative expression of ten transcripts by qRT-PCR (Fig. 7). The qRT-PCR results of *circRNA91*, *circRNA388*, *NC_040278.1_37507*, *NC_040253.1_5757*, *NC_040278.1_37602*, *NC_040262.1_21536*, *NC_040255.1_11631*, *AKT3*, *PCK1* and *PPP2R5A* were consistent with the transcriptome sequencing results.

## Discussion

In recent years, there are many reports on the role of circRNA in tumors [48, 49]. Energy metabolism is important in the occurrence and development of tumor. The lipogenesis is a major marker of tumor metabolism, because fatty acids are required for cell membrane replication and acute fat loss is a worsening wasting syndrome in patients [50, 51]. Therefore, circRNA plays an important role in regulating the fat production and decomposition. However, there is relatively few researches on the deposition of circRNA in subcutaneous fat tissues of sheep. Existing studies mostly focused on model animals such as mice, or pigs with similar genes as humans. In this study, the expression of circRNA in subcutaneous fat tissues of Duolang and Small Tail Han sheep was analyzed. The aim was to identify potential circRNA associated with adipogenic differentiation and lipid metabolism. A total of 141 differentially expressed circRNAs were identified in subcutaneous fat tissues. CeRNA network construction for differentially expressed circRNAs. The host genes and target genes of these differentially expressed circRNAs were subjected to GO and KEGG enrichment analysis. Finally, the sequencing reliability of the ten transcripts was further verified by qRT-PCR. The regulatory effects of circRNAs in fat deposition and lipid metabolism were studied to provide new evidence for the treatment of obesity and metabolic syndrome as well as for improving the quality of mutton.

The host genes of differentially expressed circRNAs were subjected to functional enrichment analysis, and significantly enriched in the pathway, such as MAPK signaling. Existing studies show that MAPK signaling pathway is highly conserved, and plays a key role in the process of cell differentiation, proliferation and apoptosis [52]. Also associated with adipocyte differentiation and fat metabolism. As a member of the mitogen-activated protein kinases (*MAPK*) family, *P38MAPK* is also a transfer station for many signal transduction pathways in cells. It is downstream regulators of β3-AR in browning of 3T3-L1 white adipocytes [53]. *MiR-29b/29c* are highly expressed in insulin-sensitive, miR-29a as the most upregulated miRNA across in insulin-sensitive tissue. They affected the MAPK signaling pathway by targeting *CTRP6*, thereby inhibiting the proliferation of porcine subcutaneous and intramuscular fat [54]. In this study, the differentially expressed circRNAs play a regulatory role in the subcutaneous fat deposition process of Duolang and Small Tail Han sheep through the MAPK signaling pathway.

Studies have shown that circRNAs cannot directly encode proteins. However, they can affect biological function by regulating mRNAs level, and some circRNAs can act as molecular sponges for adsorbing miRNAsn [55, 56]. In this paper, the differentially expressed circRNAs were predicted to target genes with differentially expressed miRNAs. The results show that a total of 36 miRNAs interacted with 71 circRNAs. Among them, *circRNA812* stably binds to miRNAs, and can target regulate *oar-miR-21*. The regulatory effect of *oar-miR-21* on fat tissues has been reported. *MiR-21* significantly promoted the differentiation of preadipocytes, and increased the expression of adiponectin in the differentiation process of 3T3-L1 adipocytes [57]. Adiponectin is a marker protein of adipose expression and secretion in adipocyte differentiation. Knockout of *miR-21* in hepatocytes of mice fed with a high-fat diet changed insulin sensitivity and the expression of a variety of key transcription factors regulating lipid and glucose metabolism [58]. NAFLD is a liver disease with metabolic disorders related to obesity and diabetes, *miR-21* can also regulate triglyceride and cholesterol metabolism in NAFLD by targeting *HMGCR* [59]. *Oar-miR-21* also plays an important role in the generation of fat in sheep.

In this study, *NC_040253.1_5757*, as an important node, plays an important role in the regulatory network of ceRNA. Through GO and KEGG enrichment analysis of target mRNAs of miRNAs, we found that mRNAs were significantly enriched in multiple items related to fat metabolism. Among them, *PPP2R5A* was significantly enriched in PI3K-Akt signaling pathway and AMPK signaling pathway. The PI3K-Akt signaling pathway is involved in the regulation of basic cellular processes such as cell growth, transcription, translation, cell proliferation, cell movement, and glycogen metabolism [60]. Many studies show that PI3K-AKT signaling pathway can regulate the production of fat by enhancing the expression of the adipogenic genes *PPARγ* and *C/EBPα*. If the pathway is abnormal, it may also lead to diseases, such as cancer and diabetes. PI3K/Akt signaling pathway can reduce liver glucose production and glycogen decomposition, increase the synthesis of glycogen and fatty acids, and store energy for other tissues [61]. PI3K/Akt was activated in inguinal subcutaneous white fat treated with phytol, and the phosphorylation level of *FoxO1* downstream target of PI3K/Akt was significantly increased. The inactivation of *FoxO1* was helpful to increase the expression of *PPARγ* and *C/EBPα*. The results show that the activation of PI3K/Akt signaling pathway can induce an increase in the number of adipocytes [62]. As an important kinase regulating energy homeostasis, AMPK is a key protein involved in a variety of signal transduction pathways. AMPK can inhibit lipogenesis, and is activated in the low-energy state of cells. This in tum restores cellular energy homeostasis by triggering the catalytic process to produce ATP and inhibiting the metabolic process that consumes ATP [63]. As a member of the adipocytokines, lipocalin plays a crucial role in systemic energy homeostasis by stimulating AMPK. ADIPOR1 and ADIPOR2 are the two main receptors for lipocalin and play key roles in metabolic pathways that regulate glucolipid metabolism, inflammation and oxidative stress. Studies show that its target genes *AMPKβ1* and *AMPKα1* were up-regulated after down-regulating the expression of liver *miR-802*. It activated the AMPK signaling pathway and increased the phosphorylation level of AMPK, resulting in decreased liver lipogenesis [64]. Studies on the porcine intramuscular fat show that the LKB1/CaMK2-AMPK-ACC1-CPT1A axis dominated the activity of AMPK signaling pathway. The activation of AMPK signaling pathway played a positive role in reducing intramuscular fat deposition [65]. AMPK has also been considered to be an antagonist of mTOR. Tang et al. [66] found that AMPK signaling was inhibited in a high ammonia environment, and the expressions of *AMPK* and *p-AMPK* were decreased. This can directly regulate lipid metabolism or indirectly through mTOR. In conclusion, AMPK signaling pathway plays an important role in the fat deposition process. Therefore, circRNA plays a regulatory role in the fat deposition process through PI3K-Akt signaling pathway and AMPK signaling pathway.

This study also have some limitations. The circRNAs, miRNAs, and mRNAs were predicted computationally, but the specific mechanisms of fat deposition regulation by these circRNAs still need to be verified experimentally.

## Conclusions

We conducted high-throughput sequencing on subcutaneous fat tissues of Duolang and Small Tail Han sheep and revealed the expression profile of circRNA and its potentialrole. Based on enrichment analysis, the differentially expressed circRNAs host genes are mainly in MAPK and AMPK signaling pathways. The target genes were significantly enriched in PI3K-Akt and AMPK signaling pathways. Among the constructed ceRNA network, the *NC_040253.1_5757* was the common MRE of *circRNA812*, *circRNA91* and *circRNA388*, and participated the regulation of subcutaneous fat deposition. In conclusion, a series of circRNAs were identified, which have potential regulatory effects on subcutaneous fat deposition and provide molecular mechanism basis for future research .

### Supplementary Information

**Additional file 1: Supplementary Table 1.** Primer information for qRT -PCR.

**Additional file 2: Supplementary Table 2.** All circular RNAs identified in fat tissues.

**Additional file 3: Supplementary Table 3.** The differentially expressed circRNAs.

**Additional file 4: Supplementary Table 4.** The targeting relationship between mRNA and miRNA.

**Additional file 5: Supplementary Figure 1.** RNA-Seq data analysis pipeline.

## Data Availability

The RNA-Seq data were submitted to the NCBI’s SRA database (Accession number: PRJNA801884 and PRJNA752700).
