# Supplementary figures and images for: Functional analysis of differentially expressed circular RNAs in sheep subcutaneous fat
Source: BMC Genomics. 2023 Oct 5;24:591. doi: 10.1186/s12864-023-09401-6 (PMC10557293; doi:10.1186/s12864-023-09401-6)

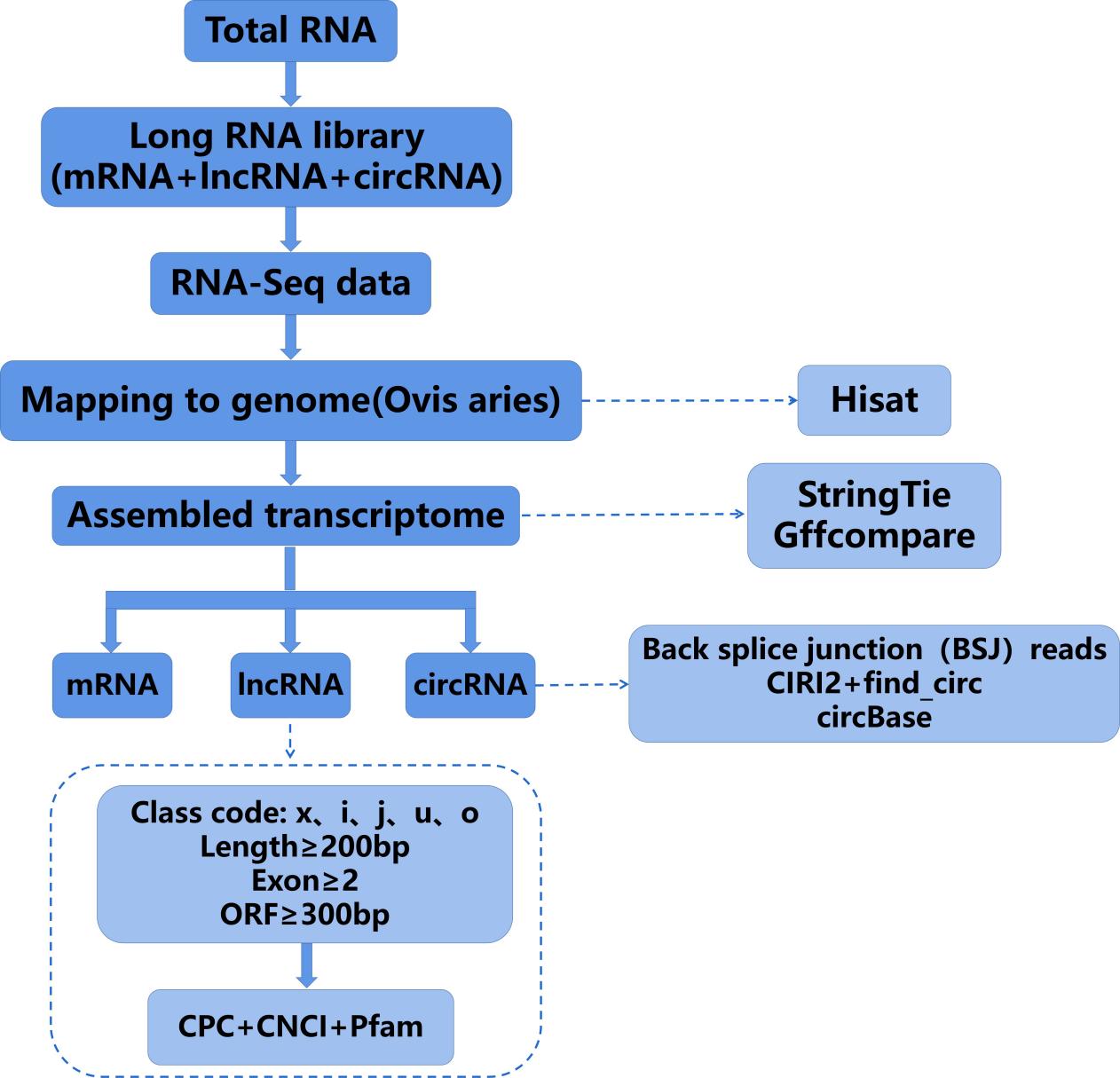


Supplementary Figure 1 | RNA-Seq data analysis pipeline.

Supplement: Supplementary file 5 — Additional file 5: Supplementary Figure 1. RNA-Seq data analysis pipeline. [file 12864_2023_9401_MOESM5_ESM.docx]
